# Effects of interprofessional education on teamwork and on knowledge chronic conditions management*

**DOI:** 10.1590/1518-8345.3095.3203

**Published:** 2019-10-28

**Authors:** Heloíse Fernandes Agreli, Marina Peduzzi, Mariana Charantola Silva, Renata Cristina Ventura Mascarelle, Pilar Espinoza

**Affiliations:** 1 University College Cork, School of Nursing and Midwifery, Cork, Ireland.; 2 Universidade de São Paulo, Escola de Enfermagem, São Paulo, SP, Brazil.; 3 Prefeitura Municipal de Campinas, Secretaria de Saúde, Campinas, SP, Brazil.; 4 Prefeitura Municipal de Embú das Artes, Secretaria de Saúde, Embú das Artes, SP, Brazil.; 5 Universidad San Sebastian, Facultad de Ciencias para el Cuidado de la Salud, Santiago, Chile.

**Keywords:** Chronic Diseases, Continuing Education, Interdisciplinary Health Team, Primary Health Care, Interprofessional Relations, Nursing, Doenças Crônicas, Educação Continuada, Equipe Interdisciplinar de Saúde, Atenção Primária à Saúde, Relações Interprofissionais, Enfermagem, Enfermedades Crónicas, Educación Continua, Equipo Interdisciplinario de Salud, Atención Primaria a la Salud, Relaciones Interprofesionales, Enfermería

## Abstract

**Objective:**

Evaluate the effect of interprofessional education on the climate of Primary Health Care teams and on the acquisition of knowledge about management of chronic non-communicable diseases.

**Method:**

Quasi-experimental study of interprofessional education intervention. Seventeen Primary Health Care teams (95 professionals) participated in the study, of which nine teams (50 professionals) composed the intervention group and eight teams (45 participants) composed the control group. The team climate inventory scale and a questionnaire on knowledge about management of chronic conditions in Primary Health Care were applied before and after intervention. Type I error was fixed as statistically significant (p<0.05).

**Results:**

In the analysis of knowledge about management of chronic conditions, the teams that participated in the interprofessional education intervention presented higher mean post-intervention increase than the teams of the control group (p < 0.001). However, in the analysis of both groups, there was no significant variation in the teamwork climate scores (0.061).

**Conclusion:**

The short interprofessional education intervention carried out during team meetings resulted in improved apprehension of specific knowledge on chronic conditions. However, the short intervention presented no significant impacts on teamwork climate.

## Abstract (pt)

**Objetivo:**

Avaliar o efeito da educação interprofissional no clima de equipes da Atenção Primária à Saúde e na apropriação de conhecimentos sobre o manejo das condições crônicas não transmissíveis.

**Método:**

estudo quase-experimental de intervenção de educação interprofissional. Participaram 17 equipes da Atenção Primária (95 profissionais), das quais nove equipes (50 profissionais) compuseram o grupo-intervenção e oito equipes (45 participantes), o grupo-controle. A escala de clima de equipe e um questionário referente aos conhecimentos sobre o manejo das condições crônicas na Atenção Primária foram aplicados antes e após a intervenção. Fixou-se erro tipo I como estatisticamente significativo (p < 0,05).

**Resultados:**

na análise dos conhecimentos sobre manejo das condições crônicas, as equipes que participaram da intervenção de educação interprofissional apresentaram aumento médio pós-intervenção maior do que as equipes do grupo-controle (p < 0,001). Contudo, na análise de ambos os grupos, não houve variação significativa nos escores de clima do trabalho em equipe (0,061).

**Conclusão:**

intervenção de educação interprofissional curta realizada durante as reuniões de equipe resultou em melhoria na apreensão dos conhecimentos específicos sobre as condições crônicas. Entretanto, a intervenção de curta duração não apresentou impactos significativos no clima do trabalho em equipe.

## Abstract (es)

**Objetivo:**

Evaluar el efecto de la educación interprofesional en el ambiente de los equipos de Atención Primaria de Salud y en la apropiación de conocimientos sobre el manejo de las condiciones crónicas no transmisibles.

**Método:**

Estudio cuasi-experimental de intervención educativa interprofesional. Participaron 17 equipos de Atención Primaria (95 profesionales); de estos, nueve equipos (50 profesionales) formaron el grupo de intervención, y ocho (45 participantes) el grupo de control. Se aplicaron antes y después de la intervención una Escala sobre el ambiente del personal y un cuestionario sobre los conocimientos acerca del manejo de condiciones crónicas. El error tipo I se consideró como estadísticamente significativo (p<0,05).

**Resultados:**

En el análisis de conocimientos acerca del manejo de condiciones crónicas, los equipos que participaron en la intervención educativa interprofesional tuvieron un aumento promedio en el posintervención superior a los equipos del grupo de control (p <0,001). Sin embargo, en el análisis de ambos grupos, no hubo variación significativa en los puntajes en el ambiente de trabajo en equipo (0,061).

**Conclusión:**

La intervención de educación interprofesional a corto plazo realizada durante las reuniones del equipo mejoró la comprensión de los conocimientos específicos sobre las condiciones crónicas. Pero no tuvo el mismo impacto significativo en el ambiente de trabajo en equipo.

## Introduction

According to the World Health Organization^(^ 1 ^)^, Chronic Non-Communicable Diseases (CNCDs) are responsible for 41 million deaths annually. The most frequent include cardiovascular diseases (17.9 million), cancers (9 million), and Diabetes Mellitus (1.6 million). This is a global problem, in which only 25% of people with CNCDs are provided care and among them only about half reach the desired goals of clinical treatment. This result is mainly due to insufficient access to health care and inadequate management of CNCDs^(^ 1 ^)^. As a way of addressing this, the ten priority recommendations of the Pan American Health Organization^(^ 2 ^)^ to improve the quality of care for CNCDs feature the reorganization of health workers into teams, ensuring interprofessional education and continuous training on the management of CNCDs.

Teamwork and collaborative practice are highlighted when considering their potential for change in light of the fragmented care and also as a means for establishing integrated health systems, which are characterized by collaboration-based forms of action^(^ 3 ^)^. In recent decades, the literature and the public policies for implementation of teamwork have conducted a broader discussion, which associates teamwork with interprofessional practice and education (IPE)^(^ 4 ^)^.

IPE is understood as an educational intervention or action aiming to improve collaboration in the care provided to users, in which members of more than one health profession learn together interactively, with the explicit purpose of improving the quality of care. This interaction in the IPE requires active participation and exchange of knowledge between different professional areas^(^ 5 ^)^. The subject gains prominence from 2010, with international publications that point to IPE as a component of a wide reform in the vocational training and health care model, aimed to work in health care networks and training for person-centered collaborative practice^(^ 3 ^,^ 6 ^)^.

Cochrane literature review on the effects of IPE on professional practice and health outcomes^(^ 5 ^)^ found fifteen studies that met the inclusion criteria, comparing IPE interventions with no educational intervention. The review reports relevant conclusions: positive effect of IPE on the areas of diabetes care, emergency departments and patient satisfaction; collaborative behavior in teamwork in a surgical center and reduction of clinical errors. The results indicate that IPE interventions can improve processes and outcomes in health care and processes in teams; however, they do not allow generalizations, considering the methodological limitations found. The authors suggest the need for studies that enable understanding the processes of changes in health care practices related to IPE^(^ 5 ^)^.

Studies of IPE interventions in the context of health care, including in the care of chronic conditions, report positive impact of IPE on satisfaction with care^(^ 5 ^,^ 7 ^)^ and on team collaboration^(^ 8 ^)^.

Considering the growing trend of health care based on collaborative teamwork and the necessary professional enhancement to provide integrated care for CNCDs, it is necessary to understand how IPE can contribute to effective teamwork, interprofessional collaboration and specific professional knowledge on the management of CNCDs in Primary Health Care (PHC). However, studies on the subject are mostly found in the international literature, from developed countries such as Australia, Belgium, Sweden, the United Kingdom and the USA^(^ 9 ^)^ and little is known about the impact of IPE interventions in developing countries such as Brazil^(^ 10 ^)^.

The results of the study will enable advancement in the proposition of an IPE program aimed at the development of integrated practices in the care of chronic conditions and the improvement of teamwork and collaborative practice. Seeking to contribute to the advancement of knowledge on the subject, this research aimed to examine the effect of IPE on the climate of PHC teams and on the acquisition of knowledge about the management of chronic conditions, using an interprofessional education course on teamwork and care for CNCDs.

## Method

Quasi-experimental study^(^ 11 ^)^ carried out in a city in the metropolitan region of São Paulo (Brazil), with a population of 245,148 inhabitants and a coverage of the Family Health Strategy (FHS) around 29%. The municipality has 15 Basic Health Units (BHU) and 19 family health teams.

The population and sample consisted of professionals working in the FHS, including administrative technicians and managers, who accepted the invitation to participate in the research and met the following inclusion criteria: 1) belonging to complete parametric teams of the FHS according to the recommendations of the Ministry of Health that is, composed of: physician, nurse, nursing auxiliary or technician, community health agents and oral health professionals: dental surgeon, oral health auxiliary or technician and 2) having worked in the same team for at least six months. As exclusion criterion, we decided to exclude from the sample professionals who were on vacation, who were on leave of absence and who could not participate in the two steps of application of the research questionnaire.

Seventeen teams belonging to ten BHUs with FHS met the inclusion criteria and the teams were allocated into the control and intervention groups by simple drawing of the units. The first five BHUs drawn were defined as intervention group and participated in the educational interventions. The other five BHUs were defined as control group and did not participate in the educational interventions.

Data collection occurred during the family health team meetings. To obtain the data, we used a self-administered instrument designed to be answered before and immediately after the IPE workshops. The instrument consisted of items related to socio-demographic data, knowledge of professionals as to the Ministry of Health recommendations for management of chronic conditions in PHC, and teamwork climate.

To evaluate the knowledge on the management of chronic conditions in PHC we used a test composed of ten questions, which dealt with the strategies recommended by Brazilian Ministry of Health^(^ 12 ^)^for care of people with chronic disease. The questions were developed by the authors of the study jointly with specialists in PHC. The topics addressed in the questions were: principles and guidelines for organization of the health care network for people with chronic diseases; preventive interventions for the population; risk factors associated with chronic conditions and forms of prevention; humane care; improvement and/or implementation of activities aimed at care for people with chronic disease and supported self-care.

Team climate was studied with application of the Team Climate Inventory (TCI) validated in the context of PHC of the SUS, with Cronbach’s alpha of 0.92^(^ 13 ^)^. The scale consists of 38 items grouped into four factors: participation in the team (12 items), support for new ideas (eight items), team goals (11 items) and task orientation (seven items) and the items are presented in Likert-like scale format. The four factors refer to the following aspects of the climate:

- Participation in the team: it concerns the participation of team members in decision making, to what extent each professional feels safe to expose their perceptions, ideas, suggest alternatives and participate in joint decisions.
- Support for new ideas: refers to support for individual members and team initiatives to introduce new or improved modes of executing work activities.
- Team goals: refers to the way goals are defined and shared among team members.
- Task Orientation: refers to the individual responsibility of each member and of the team in the execution of activities aiming at excellence in the work done. It is characterized by assessments, critical analysis and other forms of control and analysis of the performance of tasks.

Two researchers performed the data collection and educational interventions. The instrument, containing a questionnaire on sociodemographic data, knowledge test and TCI, was distributed to the subjects who answered it individually during the working hours. For the intervention group, the instrument was applied at two times: 1) Pre-test, on the first day of the IPE intervention, before the beginning of the specific activities and 2) post-test, 60 days after the pre-test and at the end of the IPE intervention. For the control group teams, the pre and post-tests were applied observing the same interval of 60 days applied to the teams that participated in the IPE intervention.

The intervention occurred through two IPE workshops with duration of 3 hours each, during the period reserved for team meetings, at their BHU. The workshops dealt with “teamwork and collaborative practice in the management of chronic conditions in PHC” and were devised based on the techniques of experiential learning theory^(^ 14 ^)^. Experiential learning consists in an approach on adult development, in particular, professional development. Professional practice is a permanent course of experiential learning, based on the following principle that all prospective professional development stems from current learning, as well as the already constituted development is indispensable for learning^(^ 14 ^)^.

The first workshop had the following objectives: 1) contextualizing the participants about teamwork and collaborative practice to provide care to people with CNCDs; 2) discussing how the team climate aspects can influence the resolution of complex cases of people with CNCDs; 3) discussing the risk factors associated with chronic conditions, preventive interventions, the three pillars of self-care and the focus on the needs of users with CNCDs using a case study as reference; 4) discussing the team goals, sharing of responsibilities and monitoring of cases of people with CNCDs.

The didactic strategy adopted in the first workshop was dialogued exposition, dynamic activity on teamwork and case study of Mr. João Adamastor, proposed in Supplement no. 35 of Primary Health Care^(^ 12 ^)^. The participants were invited to reflect on a team goal related to the management of CNCDs and the opportunities existing in the team to share responsibilities in the monitoring of cases of people with CNCDs, such as Mr. João Adamastor. At the end of the activity, the team was invited to propose ideas for improving such aspects (common objectives and task orientation).

The second workshop occurred 60 days after the first, so that the team had time to process the experience and reflect on the aspects discussed in the first workshop. It had the following objectives: 1) resuming the aspects discussed in the first workshop with team reflection on possible outcome of the case of Mr. Adamastor and 2) discussing the care strategies established by the Ministry of Health and the principles and guidelines of the Health Care network for people with chronic conditions.

The didactic strategy adopted was debate and dramatization of the service provided to Mr. Adamastor in “real” and “ideal” scenarios. The final activity consisted in the preparation of proposals in the teams to improve the management of CNCDs.

After the end of the research, the data were presented to the Municipal Department of Health and educational intervention planning was offered to the control group.

To evaluate the data, there was descriptive analysis of the characterization, knowledge about CNCDs, and teamwork climate variables through mean, standard deviation and absolute frequencies. In order to assess teamwork climate, we calculated the total score in the four factors of the TCI for each of the professionals interviewed. Then, the corresponding mean scores were calculated per team. The same procedure was followed in analyzing scores in the questionnaire on knowledge about the management of chronic diseases, that is, initially we calculated the individual score and subsequently the mean score per team. Thus, the analysis presented in the present study refers to the teams.

To analyze the correlation between the time working in the team and the scores, Pearson’s correlation coefficient was used before and after the educational intervention. Subsequently, a mixed effects model was adjusted to compare the performance of the groups (control and intervention) before and after the intervention in the scales, controlling by the time working in the team. The choice of analyzing the time working in the team is justified by the fact that in a previous study in the teams in the same municipality, a significant relation was found between time in the team and team climate scores^(^ 9 ^)^.

The data were entered into Windows Excel spreadsheets and analyzed by the Statistical Package for Social Sciences® 22 software. In all statistical tests, the significance level adopted was 5%. Internal consistency was checked through Cronbach’s Alpha coefficient.

The research was conducted between June and December 2017, after approval of the Research Ethics Committee of the School of Nursing of the University of São Paulo with opinion no. 2.125.480 and CAAE: 66504017.8.0000.5392. The participants were invited individually in the BHU to participate in the study. All invited professionals were informed on the ethical aspects and methodological design of the research. All participants were informed about the condition of control and intervention group. When they accepted to participate in the study, the professionals signed the informed consent form. The research was authorized by the Municipal Department of Health and by the managers of the BHUs. The Department of Health allowed the participation of the permanent education coordinator of the municipality to monitor the educational interventions in the BHUs. Through this participation, the permanent education coordination can become aware of the subject of teamwork climate and of the educational strategy adopted, in order to keep the educational strategy in practice and improve it according to the context.

## Results

The results related to the characterization of health professionals who participated in the IPE workshops in the BHUs are presented in Table 1. Of a total of 95 participants in the study, 50 belonged to the intervention group and 45 belonged to the control group. The sample consisted mainly of women (85%), community health agents (CHA) 57%, with complete secondary education 68%.

**Table 1:** Sociodemographic characteristics and time working in the team. Metropolitan Region of São Paulo, SP, Brazil, 2017

Characteristic		IG*		CG^†^		Total	
		(n=50)		(n=45)		(n=95)	
		n	%	n	%	n	%
Sex	Female	46	92,0	35	77,8	81	85,3
	Male	4	8,0	10	22,2	14	14,7
Training	Nursing	9	18,0	9	20,0	18	18,9
	Dentistry	2	4,0	3	6,7	5	5,3
	Medicine	1	2,0	2	4,4	3	3,2
	Another degree	4	8,0	1	2,2	5	5,3
	Postgraduate degree in Health	9	18,0	13	28,9	22	23,2
	Nursing Technician	4	8,0	4	8,9	8	8,4
	Oral Health Technician	0	0,0	2	4,4	2	2,1
	Secondary education	30	60,0	24	53,3	54	56,8
Time working in the team	< 5 years	20	40,0	27	60,0	47	49,5
	5–10 years	25	50,0	15	33,3	40	42,1
	10–15 years	4	8,0	3	6,7	7	7,4
	15–20 years	1	2,0	0	0,0	1	1,1

* IG= Intervention Group;

† CG = Control Group

The intervention group and the control group showed different sociodemographic characteristics, except for the fact that in both groups there was a predominance of female participants. In the control group there was a higher percentage of professionals with post-graduate degree (28.9%) over 18% in the intervention group. The intervention group consisted mostly of staff members with complete secondary education (60%).

With regards to the time working in the same team, the intervention group consisted mainly of professionals with 5 to 10 years of experience in the same team (50%), while in the control group, most participants reported less than 5 years of experience in the team.

Table 2 shows the percentages of correct answers at the pre- and post-intervention times, referring to the items of knowledge about the management of CNCDs and factors for assessment of teamwork climate.

**Table 2:** Analysis of variance in knowledge about the management of CNCDs* in the four factors of the TCI^†^. Metropolitan Region of São Paulo, SP, Brazil, 2017

	Pre test		Post test		p-values			
	Mean	SD^‡^	Mean	SD^‡^	Group	Step	Interaction	Time
Knowledge about the management of CNCDs*					0,012	<0,001	< 0,001	0,096
Intervention Group	4,96	1,90	7,06	1,77				
Control Group	4,84	1,78	5,42	1,53				
Participation in the team					0,051	0,068	0,458	0,942
Intervention Group	49,78	5,78	48,42	5,56				
Control Group	47,16	6,24	46,58	6,49				
Support for new ideas					0,029	0,401	0,255	0,835
Intervention Group	32,02	4,44	31,14	4,02				
Control Group	29,82	3,70	29,96	4,90				
Team goals					0,541	0,835	0,733	0,513
Intervention Group	58,78	10,37	58,92	8,36				
Control Group	57,91	7,52	57,38	9,59				
Task Orientation					0,543	0,004	0,407	0,519
Intervention Group	38,36	6,55	36,62	7,10				
Control Group	38,38	7,67	35,31	8,57				
TC^§^ total					0,175	0,061	0,961	0,956
Intervention Group	178,94	22,05	175,10	21,48				
Control Group	173,27	18,90	169,22	26,77				

* CNCDs = Chronic Non-Communicable Diseases;

† TCI = Teamwork Climate Inventory;

‡ SD = Standard Deviation;

§ TC = Teamwork Climate

Comparison between the groups shows that the intervention group presented a higher increase in mean knowledge than the control group as seen in the p-value of the interaction.

In the evaluation of teamwork climate, the interaction effect measures whether the difference in pre and post means is equal in both groups, which effectively evaluates the impact of the IPE intervention (Table 2). The interaction effect on the model measures this difference, but there was no evidence that it was significant, both in the global analysis of climate (p=0.961) and in the analysis by the factors participation in the team (p=0.458), team goals (p=0.733), support for new ideas (p=0.255) and task orientation (p=0.407).

Comparison between the intervention group and control group, regardless of the step of the study (main effect of the step, Table 2) showed that the difference between their scores was not significant (p=0.175), that is, it was not possible to differentiate the groups regarding the teamwork climate. Comparison between the control group and intervention group scores for each TCS factor showed that the intervention group teams presented significantly higher means only in the support for new ideas factor (p=0.029).

By comparing the change in means between the pre- and post-test phases, it was found that the variation in scores was not significant, both in the global analysis of the TCI scores (0.061) and in the analysis by the factors participation in the team (0.068), support for new ideas (0.401) and team goals (0.835). Only in the task orientation factor there was a significant decrease in scores for both the control group and intervention group (p=0.004). The results suggest that the adopted IPE intervention produced no effect on teamwork climate.

## Discussion

This study aimed to examine the effect of IPE on the climate of PHC teams and on the acquisition of knowledge on the management of CNCDs. The results confirmed, partially, the initial hypothesis that IPE is associated with improved knowledge on the management of chronic conditions and improved teamwork climate. The hypothesis is confirmed partially because the teams participating in IPE intervention showed significant improvement only in scores related to knowledge on management of CNCDs, but not in teamwork climate.

The results are consistent with a systematic review that describes a trend in IPE studies: the existence of evidence on the positive impact of IPE on acquisition of specific knowledge, but not on changes in attitudes and perceptions of professionals^(^ 8 ^)^, as measured by the TCI.

The literature^(^ 9 ^)^ presents evidence of positive impact of IPE on interprofessional attitudes/perceptions and specific knowledge, but little is known about the impact of IPE on professional behaviors, patient outcomes and interprofessional practice.

In order to understand the impact of IPE on professional practice, there are quasi-experimental and practical intervention studies aimed at improving interprofessional work and quality in health care.

Evidence from quasi-experimental studies shows positive impact of IPE interventions on the improvement of interprofessional work^(^ 15 ^-^ 16 ^)^. However, the systematic review on the effect of interprofessional interventions on practice presents methodological weaknesses and lack of conclusive findings regarding the impact of such interventions on the improvement of interprofessional work and health care^(^ 17 ^)^.

The results of this study corroborate the findings of other studies that aimed to evaluate the impact of short IPE intervention on the acquisition of specific knowledge. On the subject of management of CNCDs and interprofessional work, we highlight the controlled experimental study for evaluation of IPE intervention^(^ 18 ^)^. According to the authors, a short intervention (11 hours) on specific content can positively impact both the self-perception of the capacity to manage CNCDs and the attitudes related to interprofessional work. The potential of short IPE intervention can positively influence the attitude towards interprofessional work^(^ 19 ^)^. However, short IPE interventions (less than 2 hours and a half) show no potential for change in professional knowledge^(^ 20 ^)^.

The findings related to teamwork climate differ from those of a previous study related to IPE^(^ 21 ^)^ in which increased TCI scores were found after IPE intervention. The main differences between this study and the aforementioned study on IPE^(^ 21 ^)^ consist in the duration of the IPE intervention, the interval between pre and post test, and the existence of a control group. In an initial sample of 79 participants, the authors performed an IPE intervention with 12 hours of duration, with post test at four and eight months after intervention and did not include control group. Although the aforementioned study^(^ 21 ^)^ suggests that 12-hour interventions over eight months of duration are capable of positively impacting the teamwork climate, the implementation of an IPE intervention involving professionals in the context of clinical practice may pose challenges^(^ 17 ^)^, especially in the context of shortage of staff, which may compromise the participation in professional education activities.

As for the team climate scores, it is worth noting that the only aspect that showed significantly decreased scores, both for the control group and intervention group, was the task orientation factor. This involves reflection as a key element for the existence of interprofessional negotiations, clarity about the responsibilities and roles of each professional in the team^(^ 22 ^)^.

The significantly decreased scores in the task orientation factor suggests that when answering the questionnaire the second time the professionals presented a more negative perception exactly in the factor that translates the team’s capacity to reflect on their work in pursuit of excellence.

The time working in the team was not related to the teams’ knowledge about the management of CNCDs, and showed no effect on teamwork climate scores. The time of experience in the same team is described in the literature as “team stability,” this is an attribute that stimulates shared work and joint decision-making^(^ 23 ^)^.

Finally, if on the one hand short IPE interventions showed to be effective to improve knowledge about the management of CNCDs, on the other hand they did not contribute to improve the teamwork climate. This result refers to a reflection on the characteristics of teamwork climate, which receives contributions from social interactions, relationships between professionals from different areas, and influence of organizational culture^(^ 24 ^)^, complex aspects that are less sensitive to a short intervention restricted to the scope of the teams.

Collaborative practice and teamwork climate^(^ 25 ^)^ involve relational, organizational, contextual aspects and some tensions, for example, the development of collaborative competencies versus autonomy of each profession. IPE interventions aimed at improving team climate should consider such tensions, which can be challenging, especially in a short period of time. The tensions that exist in practice do not necessarily need to be “solved,” but they need to be confronted in order to allow the advance of the movement in favor of collaborative practice. Accordingly, the very complexity of interprofessional work and its tensions help to understand the possible reasons for the lack of impact on team climate of a short IPE intervention in an isolated way^(^ 24 ^)^.

One of the limitations of the study was the composition of the intervention and control groups. There was difficulty in obtaining participants to compose the control group, since the professionals who would not participate in the intervention showed no interest in completing the questionnaire in the pre and post test stages. There was also heterogeneity of the control and intervention groups regarding education. The control group presented more professionals with higher education, which is not appropriate for an intervention study. The fact that the intervention group is composed mostly of staff with secondary education may have interfered with the results of knowledge before and after the intervention, as well as with the team climate, due to the power relations between professionals with different levels of education.

Another limitation pointed out in the study was the non-proportionality between the sexes, which may have contributed to the results observed and the fact that the working conditions, the history of the teams, the previous training and the history of their territories may have interfered with the teams’ climate and knowledge, which were not addressed by the IPE intervention performed.

## Conclusion

IPE intervention in the format of short workshops with the use of a case study carried out with participation presents the potential to have positive impact on the knowledge about the management of CNCDs in PHC, but not on the teamwork climate.

The IPE intervention with 6 hours of duration proved to be sufficient to significantly increase the common knowledge about the CNCDs among the members of the interprofessional teams. An important facilitating factor for implementation of educational intervention was the choice of occasion for intervention: team meetings at the BHU. This factor was a facilitating one because in most BHUs all professionals of the team were able to participate, which did not occur in the municipality when the educational interventions took place outside the BHU. Other facilitating factors were the low cost of the educational intervention and the teaching experience of the educator who conducted the workshops. A barrier to the implementation of the interprofessional intervention was the fact that some professionals, especially physicians and nurses, were unable to participate in the intervention because they had scheduled appointments or had to leave during the workshops to provide care to users.

CNCDs have an important epidemiological impact on public health and require interprofessional organization of care. This study contributes with knowledge about how to develop IPE activity for interprofessional management of CNCDs in PHC. Future studies are necessary to determine whether the effects of IPE intervention are maintained over time and whether the effects of this knowledge have impact on interprofessional teamwork and collaborative practice. Due to the need to advance the proposition of IPE programs aimed at improving the teamwork climate, it is suggested that future IPE intervention studies using the quasi-experimental design. Those studies should be combined with qualitative studies and adopting interventions with duration of more than 6 hours and monitoring the results at an interval exceeding 60 days between pre and post test.

Although presenting limitations, this study was the first approach to advance in the discussion about the effect of IPE interventions on teamwork in the FHS. This is a relevant study to understand which characteristics of IPE are capable of producing positive effect on the knowledge of PHC professionals and on the improvement of teamwork. To date, little is known about which interventions should be adopted in the daily work to prepare teams for better management of CNCDs.

## References

[1] World Health Organization (2019). Noncommunicable diseases.

[2] (2015). Innovative Care for Chronic Conditions: Organizing and Delivering High Quality Care for Chronic Noncommunicable Diseases in the Americas.

[3] World Health Organization (WHO) (2010). Framework for action on interprofessional education & collaborative practice.

[4] Peduzzi M, Agreli HF (2018). Teamwork and collaborative practice in Primary Health Care. Interface - Comunicação, Saúde, Educ.

[5] Reeves S, Perrier L, Goldman J, Freeth D, Zwarenstein M (2013). Interprofessional education: effects on professional practice and healthcare outcomes (update). Cochrane Database Syst. Rev.

[6] Frenk J, Chen L, Bhutta ZA, Cohen J, Crisp N, Evans T (2010). Health professionals for a new century: transforming education to strengthen health systems in an interdependent world. Lancet.

[7] Reeves S, Zwarenstein M, Goldman J, Barr H, Freeth D, Hammick M (2008). Interprofessional education: effects on professional practice and health care outcomes. Cochrane Database Syst Rev.

[8] Yamani N, Asgarimoqadam M, Haghani F, Yamani AQA (2014). The effect of interprofessional education on interprofessional performance and diabetes care knowledge of health care teams at the level one of health service providing. Adv Biomed Res.

[9] Reeves S, Fletcher S, Barr H, Birch I, Boet S, Davies N (2016). A BEME systematic review of the effects of interprofessional education: BEME Guide No. 39. Med Teacher.

[10] Sunguya BF, Hinthong W, Jimba M, Yasuoka J (2014). Interprofessional education for whom? Challenges and lessons learned from its implementation in developed countries and their application to developing countries: a systematic review. PloS one.

[11] Eccles M, Grimshaw J, Campbell M, Ramsay C (2003). Research designs for studies evaluating the effectiveness of change and improvement strategies. Qual Saf Health Care.

[12] Ministério da Saúde (BR), Secretaria de Atenção à Saúde, Departamento de Atenção Básica (2014). Strategies for the care of person with chronic disease.

[13] Silva MC, Peduzzi M, Sangaleti CT, Silva D, Agreli HF, West MA (2016). Cross-cultural adaptation and validation of the teamwork climate scale. Rev. Saúde Pública.

[14] Kolb D (1984). Experiential learning.

[15] Körner M, Luzay L, Plewnia A, Becker S, Rundel M, Zimmermann L (2017). A cluster-randomized controlled study to evaluate a team coaching concept for improving teamwork and patient-centeredness in rehabilitation teams. PloS one.

[16] Weaver SJ, Rosen MA, Diaz Granados D, Lazzara EH, Lyons R, Salas E (2010). Does teamwork improve performance in the operating room? A multilevel evaluation. Jt Commission J Qual Patient Saf.

[17] Reeves S, Pelone F, Harrison R, Goldman J, Zwarenstein M (2017). Interprofessional collaboration to improve professional practice and healthcare outcomes. Cochrane Library.

[18] Darlow B, Coleman K, McKinlay E, Donovan S, Beckingsale L, Gray B (2015). The positive impact of interprofessional education: a controlled trial to evaluate a programme for health professional students. BMC Med Educ.

[19] Watts F, Lindqvist S, Pearce S, Drachler M, Richardson B (2007). Introducing a post-registration interprofessional learning programme for healthcare teams. Med Teacher.

[20] Olson R, Bialocerkowski A (2014). Interprofessional education in allied health: a systematic review. Medical education.

[21] Wong AKC, Wong FKY, Chan LK, Chan N, Ganotice FA, Ho JSC (2017). The effect of interprofessional team-based learning among nursing students: A quasi-experimental study. Nurse Educ Today.

[22] D'Amour D, Goulet L, Labadie JF, Martín-Rodriguez LS, Pineault R (2008). A model and typology of collaboration between professionals in healthcare organizations. BMC Health Serv Res.

[23] Slotegraaf RJ, Atuahene-Gima K (2011). Product development team stability and new product advantage: The role of decision-making processes. J Market.

[24] Hudson JN, Croker A (2018). Educating for collaborative practice: an interpretation of current achievements and thoughts for future directions. Med Educ.

[25] Agreli HF, Peduzzi M, Bailey C (2017). Contributions of team climate in the study of interprofessional collaboration: A conceptual analysis. J Interprof Care.

